# Evaluation of *Physarum polycephalum* plasmodial growth and lipid production using rice bran as a carbon source

**DOI:** 10.1186/s12896-015-0188-y

**Published:** 2015-08-01

**Authors:** Hanh Tran, Steven Stephenson, Erik Pollock

**Affiliations:** School of Biotechnology, Ho Chi Minh International University, Ho Chi Minh City, 70000 Vietnam; Department of Biological Sciences, University of Arkansas, Fayetteville, Arkansas 72701 USA; Stable Isotope Laboratory, University of Arkansas, Fayetteville, Arkansas 72701 USA

## Abstract

**Background:**

The myxomycete *Physarum polycephalum* appears to have remarkable potential as a lipid source for biodiesel production. The present study evaluated the use of rice bran as a carbon source and determined the medium components for optimum growth and lipid production for this organism.

**Results:**

Optimization of medium components by response surface methodology showed that rice bran and yeast extract had significant influences on lipid and biomass production. The optimum medium consisted of 37.5 g/L rice bran, 0.79 g/L yeast extract and 12.5 g/L agar, and this yielded 7.5 g/L dry biomass and 0.9 g/L lipid after 5 days. The biomass and lipid production profiles revealed that these parameters increased over time and reached their maximum values (10.5 and 1.26 g/L, respectively) after 7 days. *Physarum polycephalum* growth decreased on the spent medium but using the latter increased total biomass and lipid concentrations to 14.3 and 1.72 g/L, respectively.

**Conclusions:**

An effective method for inoculum preparation was developed for biomass and lipid production by *P. polycephalum* on a low-cost medium using rice bran as the main carbon source. These results also demonstrated the feasibility of scaling up and reusing the medium for additional biomass and lipid production.

## Background

Biodiesel, which can be produced by the transesterification of triglycerides, is renewable and more environmentally friendly than traditionally used fossil fuels [1]. In the past, plant oil commonly has been used as a source of lipids for biodiesel production. More recently, lipid production from a number of microorganisms, including algae, oleaginous yeasts and fungi, have been investigated as possible replacements of plant oil as a more sustainable approach to producing biodiesel, since microorganisms grow more rapidly and make much more efficient use of space. The biggest factor that has prevented biodiesel from being widely commercialized is its high cost compared with conventional fossil fuels. The raw material (oil/lipids) required for biodiesel production is responsible for about 60–75 % of the total cost of producing this type of fuel [1]. Therefore, lowering the cost of the raw material would overcome this obstacle to the wider use of biodiesel. There are several possible approaches, including screening for high lipid-producing microorganisms or genetically modifying existing ones for higher lipid production [2]. In addition, using a simple, inexpensive medium for culturing lipid-producing microorganisms also would represent a promising strategy.

*Physarum polycephalum* Schwein. is a member of the order Physarales of the class Myxomycetes, a group of fungus-like eukaryotic organisms commonly known as slime molds. Like all other members of this group, the life cycle of *P. polycephalum* is characterized by a distinctive multinucleate trophic stage (called a plasmodium). The plasmodium of *P. polycephalum* commonly occurs on decaying plant material in nature [3]. The rapid rate of growth, the absence of cell walls and the ease of culturing have resulted in the plasmodium of *P. polycephalum* being widely used in cell biology research. However, only two studies of which we are aware have examined *P. polycephalum* with respect to lipid production. Poulos and Thompson [4] found that *P. polycephalum* could undergo rapid growth and accumulate a considerable amount of lipids, and Tran et al. [5] demonstrated that the lipids derived from myxomycetes could be used to produce biodiesel. These previous reports prompted the present study.

In brief, the primary purposes of this research were first to investigate the possibility of culturing *P. polycephalum* on an agricultural waste (rice bran) and then to determine the optimum medium composition for an enhancement of biomass and lipid production using response surface methodology (RSM).

## Results and discussion

### Effect of inoculum types on plasmodial growth on the rice bran medium

This portion of the study reported herein was carried out to test the feasibility of using rice bran as the main carbon source for growing *P. polycephalum* and also to determine the suitable inoculum type for this purpose. Glucose in nutrient agar was replaced with rice bran with the same concentration (5 g/L).

**Fig. 1:**
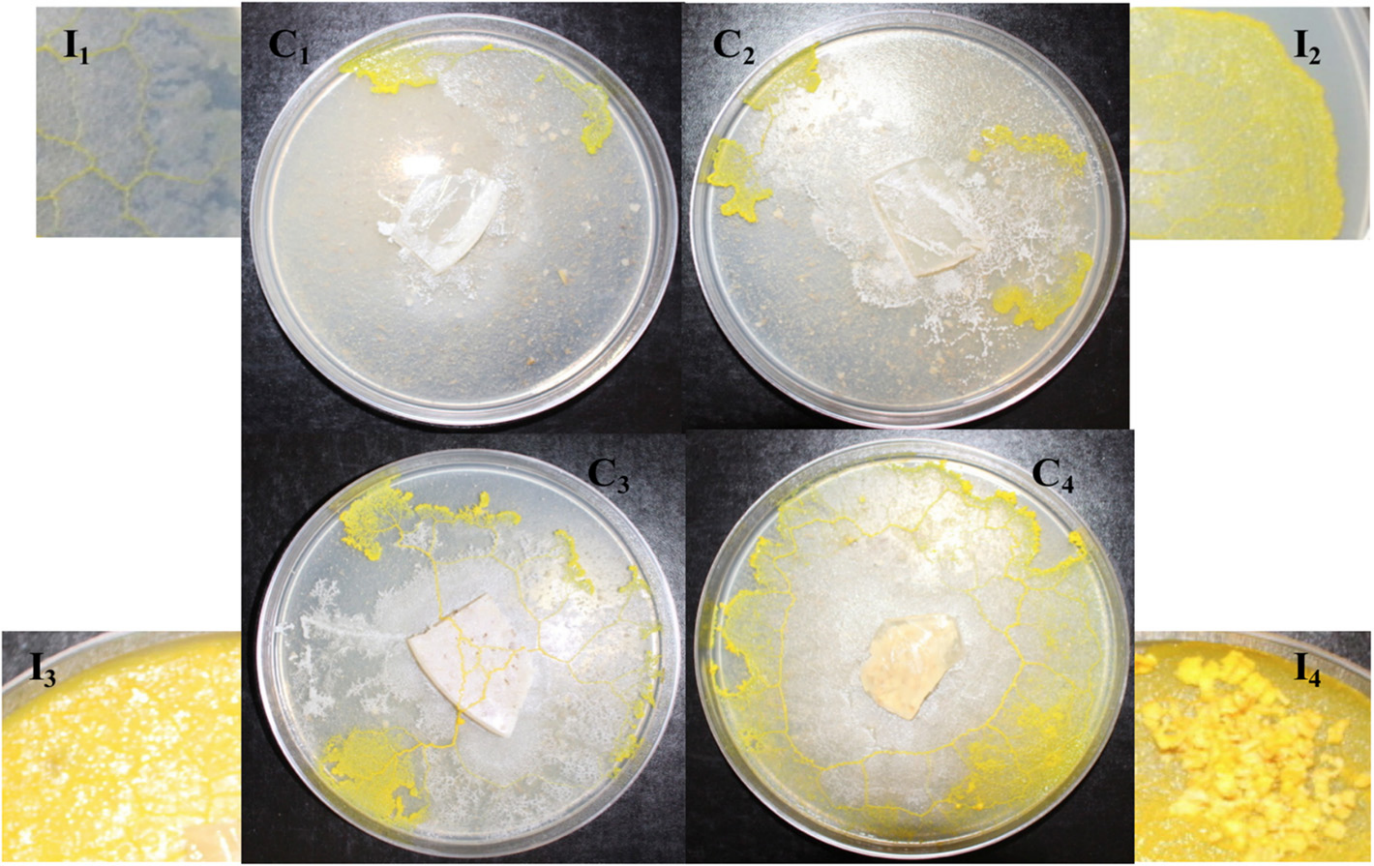
Different *P. polycephalum* inoculum types and their corresponding growth responses in various rice bran cultures. I_1_: water agar inoculum; I_2_: nutrient agar inoculum; I_3_: 1 % agar embedded with 10 % oatmeal inoculum; I_4_: nutrient agar sprinkled with oat flake inoculum. C_1_, C_2_, C_3_, and C_4_ are corresponding cultures of each inoculum

Traditionally, material taken from the margin of an actively growing plasmodium on nutrient agar/agar is used as an inoculum [6, 7]. However, it seems likely that the method for producing inoculum should be modified according to the particular purpose of the research being carried out. An agar-oat flakes plate containing 1 % agar embedded with 10 % *w/v* rolled oat flakes was used to evaluate the foraging responses of *P. polycephalum* when exposed to various food sources, including 10 % oat flakes [8]. However, these inoculum types were characterized by quite poor growth on the rice bran substrate, especially the inoculum prepared on water agar and nutrient agar without oatmeal (Fig. 1).

**Fig. 2:**
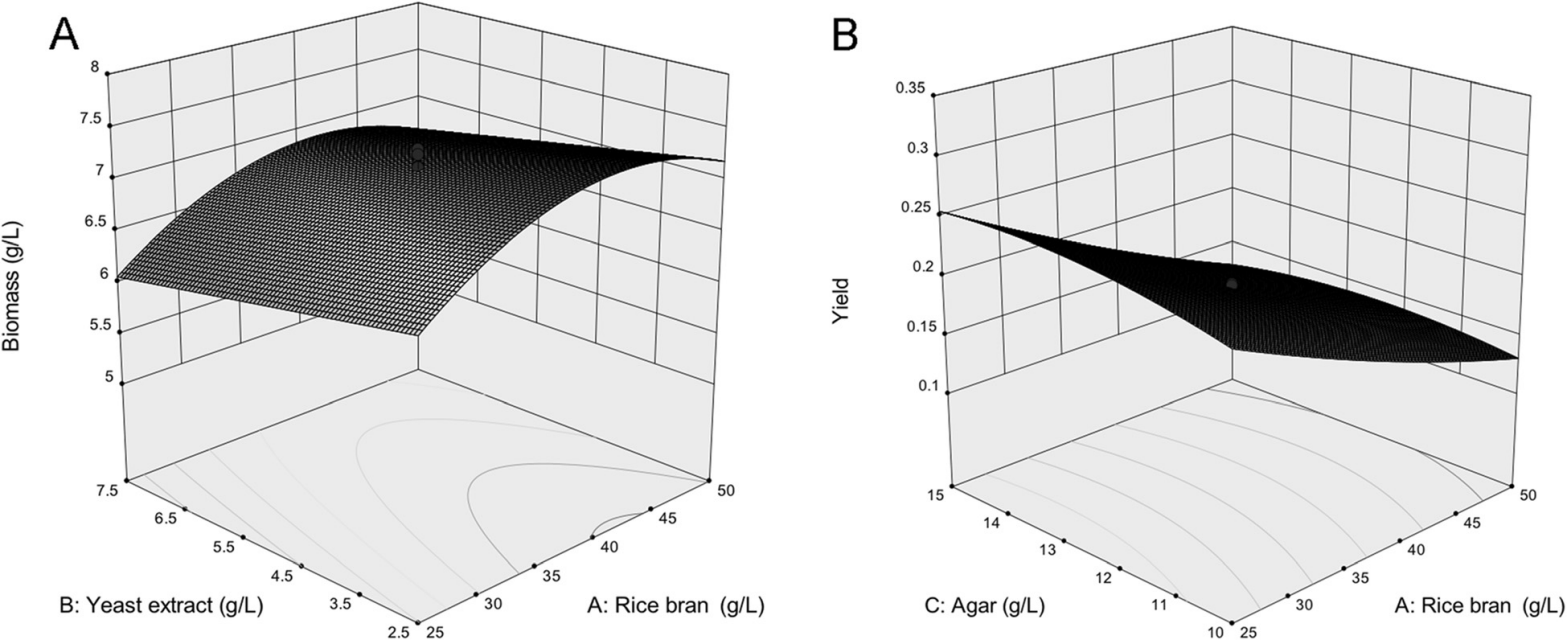
Response surface plots representing rice bran and yeast extract concentration effects on DCW (g/L) at a given agar concentration of 12.5 g/L (**a**); Representing the effect of rice bran and agar concentration on Yield at a given yeast extract concentration of 5.0 g/L (**b**)

Since rice bran is a complex substrate, it was anticipated that *P. polycephalum* would need some adaptation in order to be able to utilize this type of substrate. Based upon the fact that oat flakes commonly have been used to feed myxomycete plasmodia for high biomass production [9], we decided to culture *P. polycephalum* plasmodia on nutrient agar containing glucose as the carbon source. The culture was incubated in the dark at room temperature for two days. Oat flakes were sprinkled on surface of actively growing plasmodia. The cultures were then incubated for one more day until the oat flakes were fully covered by the plasmodium. A plug of agar (ca 2 cm^2^) bearing plasmodia associated with the oat flakes was used as the inoculum for rice bran cultures.

Based upon the data obtained in this portion of the overall study, the inoculum prepared on nutrient agar sprinkled with oat flakes resulted in a higher dry biomass production (1.5 g/L) (Table 1). Presumably, when oat flakes were sprinkled onto an actively growing plasmodium, the plasmodium excretes a set of enzymes to hydrolyze the oat flakes. As such, when this plasmodium was transferred to a rice bran culture, it could readily utilize rice bran. It should be noted that if plasmodial growth is delayed, then other microorganisms are likely to overgrow and produce negative effects for the plasmodium, a phenomenon that was observed for cultures prepared with other inoculum types, especially water agar and nutrient agar.

**Table 1:** Biomass production of cultures prepared with different inoculum types

Inoculum type	DCW (g/L)
Water agar	0.3 ± 0.01
Nutrient agar	0.5 ± 0.01
Oatmeal agar	0.9 ± 0.05
Nutrient agar sprinkled with oat flake	1.5 ± 0.2

An effort was made to verify that plasmodial growth on rice bran cultures prepared using oatmeal agar and a nutrient oat flake inoculum was not simply the result of the utilization of the remaining amount of oatmeal or oat flakes in a particular type of inoculum. These two inoculum types were transferred onto water agar plates. From our observations, very little plasmodial growth was recorded (unpublished observations). This indicated that the plasmodium does utilize rice bran effectively for growth and that the latter is not just the product of the oat flakes or oatmeal remaining in the inoculum.

It should be noted that several other carbon sources, including glucose, rice hulls, and mushroom powder (certain types of mushrooms represent a favorite substrate of *P. polycephalum* in nature) were tested along with rice bran. Glucose cultures provided the highest amount of biomass, followed by rice bran, mushroom powder and rice hull cultures. However, based on the productivity and the cost of the substrate (unpublished data), rice bran was selected for further studies.

### Effects of media composition on plasmodial growth and lipid production

The purpose of this portion of the study was to determine the optimum composition of the medium used, including the effect of agar, rice bran and yeast extract concentrations and the interactions (if any) among them with respect to biomass and lipid production and the product yield of *P. polycephalum* plasmodia. RSM was selected over the conventional method used to assess growth responses because in contrast to the conventional method, in which only a single factor is varied at any one time, RSM allows a number of factors considered in parallel. Therefore, it was possible to evaluate the effect of each variable as well as the interactions among them on the final product. In addition, RSM also requires fewer experimental trials.

The response surface analysis was based on multiple linear regressions that took into account the main, quadratic and interaction effects in accordance with the equation

$$ \mathrm{Y}={\upbeta}_0+{\displaystyle \sum {\upbeta}_i{\mathrm{X}}_i+{\displaystyle \sum {\upbeta}_{ii}{\mathrm{X}}^2+{\displaystyle \sum {\upbeta}_{ij}{\mathrm{X}}_i{\mathrm{X}}_j}}} $$

where *Y* is the predicted response, *xi* and *xj* are input variables which influence the response variable *Y, β*_*o*_ is the offset term, *β*_*i*_ is the *i*th linear coefficient, *β*_*ii*_ is the *i*th quadratic coefficient, and *β*_*ij*_ is the *ij*th interaction coefficient.

The polynomial equations in term of actual factors for DCW (g/L) (Y1), and Yield (Y2) are those listed below.

$$ {\mathrm{Y}}_1=-8.38+0.26{\mathrm{x}}_1-0.10{\mathrm{x}}_2+1.66{\mathrm{x}}_3-3.08\times {10}^{-3}{\mathrm{x}}_1^2-0.06{\mathrm{x}}_3^2 $$

$$ \begin{array}{l}{\mathrm{Y}}_2=0.01-5.67\times {10}^{-3}{\mathrm{x}}_1-0.01{\mathrm{x}}_2+0.05{\mathrm{x}}_3+1.67\times {10}^{-4}{\mathrm{x}}_1{\mathrm{x}}_2-2.07\times {10}^{-4}{\mathrm{x}}_1{\mathrm{x}}_3+7.50\times {10}^{-}\\ {}{}^4{\mathrm{x}}_2{\mathrm{x}}_3+3.87\times {10}^{-5}{\mathrm{x}}_1^2-4.00\times {10}^{-4}{\mathrm{x}}_2^2-1.76\times {10}^{-3}{\mathrm{x}}_3^2\end{array} $$

Since the lipid content (the percentage of lipid per one gram of dry biomass) of all treatments was not found to be significantly different (10–12 %), it would appear that lipid production depends entirely on DCW production. Therefore, the lipid production model has the same statistical values as those of DCW and is not listed. Results of ANOVA analysis for response surface quadratic model of DCW production and yield are listed in Table 2. In general, R^2^ (multiple correlation coefficient), CV (coefficient of variation) and *P* values are often used to evaluate the adequacy of a model. The closer the *R*^*2*^ value is to 1, the greater the correlation between the experimental and predicted values. The *P* values of DCW and Yield models were <0.05, which is clear evidence that the models are reliable. The CV value is the ratio of the standard error of the estimate to the mean value of the observed response. This value reflects the degree of precision with which the experiments are compared. A low reliability of an experiment is usually indicated by a high value of CV (>20). The CV values of the two models in this case were 5.2 for DCW model and 3.58 for Yield model, both of which are desirable.

**Table 2:** ANOVA analysis for response surface quadratic model

Variability	DCW (g/L)	Yield
R^2^ of model	0.90	0.99
Adjusted R^2^ of model	0.81	0.98
F value of model	13.63	121.74
P > F	0.034	0.0003
CV of model	5.20	3.58

The results obtained reveal that the rice bran concentration (*x*_*1*_) and yeast extract concentration (*x*_*2*_) had the most significant effects on DCW (*P* < 0.05). Agar (*x*_*3*_) had no significant effect on DCW. However, the quadratic value (*x*_*3*_^*2*^), for agar, together with the corresponding value for rice bran (*x*_*1*_^*2*^), showed significant effects on DCW. As such, there is no significant interaction among the three studied factors with respect to DCW and, by extension, lipid production (Fig. 2a).

Rice bran generally consists of about 50 % carbohydrate (including fiber, free sugar and starch), 10 % protein and small amounts of several minerals and vitamins [10]. Yeast extract contains about 11 % organic nitrogen, trace amounts of vitamins and a low concentration of carbohydrate. In contrast, agar simply provides the surface on which the plasmodium grows. Plasmodial growth increased significantly when the concentration of rice bran was increased from 25 to 40 g/L. However, when rice bran concentration exceeded 50 g/L, no additional growth enhancement was observed. This would be explained by the fact that a higher rice bran concentration would reduce the amount of free water (water availability) of the culture. Minor increased delays in plasmodial formation with higher nutrient levels (corn meal concentrations) were reported by Clark et al. [11], but there was no evidence for a general trend.

It is well known that myxomycetes feed upon bacteria and yeasts. However, from our observations, a high concentration of yeast extract promoted the growth of yeast and bacteria. When other microorganisms colonized areas of the plate, a plasmodium displayed a tendency to migrate away from these areas, resulting in the formation of less biomass and eventually poor growth. Thus, increasing yeast extract concentration has a negative effect on biomass and therefore lipid production. Although the amount of yeast extract optimized for cell growth is small (0.79 g/L), excluding yeast extract from the medium would reduce growth significantly (unpublished observations). The reason for this is unknown.

It was noted by Clark et al. [11], that decreasing the hardness of the medium (reducing agar concentration in the range of 0.9–2.3 %) slightly delays plasmodial formation of the myxomycete *Didymium iridis* (Ditmar) Fr. However, there was no evidence for any type of general trend. Lower agar concentration also was found to reduce biomass production of *P. polycephalum* in the present study, but the effect was not statistically significant. This would be explained by the fact that the agar concentrations used both by Clark et al. [11] and in the present study are still sufficient to create semi-solid or solid surfaces. As such, the plasmodium could still grow properly. We observed that at lower concentration of agar (smaller than 0.5 %), the growth of *P. polycephalum* was influenced significantly. Higher agar concentration (above 2.5 %) also had a negative effect on *P. polycephalum* (unpublished observations). In general, a higher agar concentration would reduce water availability in the culture, but a lower agar concentration would reduce the amount of oxygen. In both cases, the growth of the plasmodium would suffer.

In terms of *Yield*, three factors—including rice bran (x_1_), yeast extract (*x*_2_) and agar concentration (x_3_)—along with the quadratic values of rice bran (x_1_^2^) and agar (x_3_^2^) were found to have a significant influence on Yield values. In addition, there also was a significant interaction between rice bran and agar concentration on Yield (Fig. 2b).

The optimum medium for plasmodial growth was found to consist of 37.5 g/L rice bran, 0.79 g/L yeast extract and 12.5 g/L agar (Fig. 2). This type of medium yielded 7.5 g/L DCW and 0.9 g/L lipid after 5 days.

### Effect of incubation time on plasmodial growth and lipid production

**Fig. 3:**
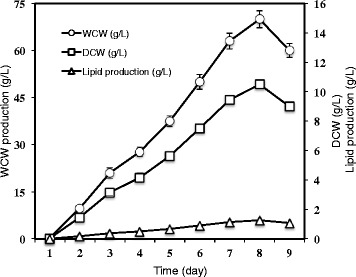
Growth and lipid production profiles for *P. polycephalum*

As noted above, the optimum medium for plasmodial growth was found to consist of 37.5 g/L rice bran, 0.79 g/L yeast extract and 12.5 g/L agar. Under these conditions, 7.5 g/L DCW and 0.9 g/L lipid were obtained after 5 days. As was the case in the RSM experiments, the developing plasmodium was collected after 5 days for analysis of biomass and lipid analysis. This portion of the study attempted to investigate the growth and lipid production profiles of *P. polycephalum* over a period of time. Sixteen cultures were prepared using the same inoculum size and medium composition as described above. Each day over a period of 8 days, the biomass of the plasmodium present was collected from two randomly selected plates. Data on wet cell weight (WCW), DCW, and lipid production were recorded and are presented in Fig. 3. Once again, incubation time had no effect on the lipid content of this particular myxomycete. The lipid content was found to vary from 10 to 12 % during the incubation period. The water content (85 %) of plasmodium also was unchanged, regardless of the incubation time. Biomass (WCW and DCW) and lipid production increased dramatically and reached maximum values of 70 g/L, 10.5 g/L and 1.26 g/L, respectively, on the seventh day. Biomass and lipid content decreased on the eighth day when the plasmodium showed signs of degradation. Therefore, the best time for biomass harvest is after 7 days. In other studies (e.g., Knowles and Carlile [6]), the plasmodium of myxomycetes has been reported to die after 8 to 10 days [6].

### Biomass and lipid production of *P. polycephalum* in the spent medium

A sterile glass container (20 cm in diam.) containing 200 ml of a rice bran medium was used for cultivation of *P. polycephalum* in this portion of the study. The plasmodium was collected after one week, after which the surface of the medium was removed by using a sterile blade. New inoculum was added and the culture was then incubated for another week and the new plasmodium was harvested at the end of this period. The data obtained are given in Table 3.

**Table 3:** Biomass and lipid production of *P. polycephalum* on fresh and spent media

Culture	DCW (g/L)	Lipid production (g/L)
First time	9.8 ± 0.23	1.18 ± 0.02
Second time	4.5 ± 0.20	0.54 ± 0.02
Total	14.3 ± 0.44	1.72 ± 0.04

The values for biomass (9.8 g/L) and lipid production (1.18 g/L) of *P. polycephalum* after 1 week in the glass container (20 cm in diam.) were not significantly different from those of the Petri dish (14 cm in diam.), for which the biomass and lipid concentrations were 10.5 g/L and 1.26 g/L, respectively. Since the volume of medium in the glass container was four times higher than that in the Petri dish, the results obtained were positive, since there was no indication that scaling up has a negative effect the growth and lipid production of this species of myxomycete.

*Physarum polycephalum* showed a noticeably slower growth rate when inoculated on the spent medium. Only 4.5 g/L DCW and 0.54 g/L lipid were obtained from the plasmodium produced on the spent medium. Presumably, this could be explained by decreased nutrient levels as well as water content in the spent medium. It also should be noted that the removal of the surface layer of the medium before adding new inoculum of *P. polycephalum* was crucial. Otherwise, *P. polycephalum* was characterized by very little or no growth. This is because removing the surface layer provides the plasmodium with access to the unexploited or less exploited lower portion of the medium. Regardless of the lower levels of biomass and lipid production in the second culture, culturing *P. polycephalum* on the spent medium increased the total biomass and lipid concentrations from 10.5, and 1.26 g/L to 14.3 and 1.72 g/L, respectively. This resulted in an increase in lipid productivity to 45.9 mg lipid/g rice bran. This productivity is comparable with that of *Mortierella isabellina* (an oleaginous fungus) in soybean hull cultures, for which productivity was 47.9 mg lipid/g soybean hull [12]. Compared with other oleaginous microorganisms, *P. polycephalum* showed a faster growth rate and higher biomass production but a lower lipid content. For example, *Metchninkowia pulcerrima,* a fungus investigated by Santamauro et al. [13], was found to produce 7.4 g/L dry biomass with a lipid content of 40 % after 15 days in glycerol culture. However, since rice bran is an agricultural waste, which is readily available, the difference in lipid production would seem to be acceptable.

Our previous study found the lipids of *P. polycephalum* to be composed mainly of triglyceride (95.5 %) and a trace amount of free fatty acids, which makes them suitable for biodiesel production [5]. In addition, myxomycete plasmodia have no cell walls, so that lipid extraction is much easier than is the case for other microorganisms (e.g., algae) [14, 15].

## Conclusions

When culturing *P. polycephalum* on a rice bran-based medium, sterile oat flakes should be added to an actively growing inoculum and incubated for 1 day before use. Rice bran and yeast extract concentrations were found to have significant effects on both biomass and yield, but agar had a significant effect only on yield. The growth and lipid production of *P. polycephalum* reached maximum values after 7 days. Despite the fact that the growth of *P. polycephalum* became noticeably slower in the spent medium, using the latter increased the total biomass and lipid concentrations to 14.3 and 1.72 g/L, respectively.

## Methods

### Materials

The strain of *P. polycephalum* used in the present study was obtained as a sclerotium from the Carolina Biological Supply Company (Burlington, North Carolina). The defatted white rice bran we used was obtained from the University of Arkansas Rice Quality Laboratory. Two types of media were used. The first was water agar (1.0 L of agar consisted of 20 g of agar and 1000 mL of water) and the second was nutrient agar (1.0 mL of the nutrient agar contained 100 mL of a basal salt solution, 5.0 g of glucose [Difco], 2.5 g of yeast extract [Difco], 20.0 g of agar, and 900 mL of distilled water adjusted to pH 5.5). The basal salt solution contained 29.78 g of citric acid, 33.10 g of K_2_HPO_4_, 2.50 g of NaCl, 1.00 g of MgSO_4_.7H2O, 0.50 g of CaCl_2_.2H2O, and 1000 mL distilled water.

### The effect of inoculum type on plasmodial growth of *P. polycephalum*

The plasmodium of *P. polycephalum* was activated by placing the sclerotium on the surface of a nutrient agar plate. Once activated, sterile oat flakes were added on the actively growing plasmodium and the latter incubated in the dark for 1 day. To prepare the different inoculum types, a plug of agar (ca 2 cm^2^) bearing a portion of the active plasmodium growing in association with the oat flakes was transferred to plates containing different media, including water agar, water agar with 10 % oatmeal, and nutrient agar. Plates were incubated in the dark at room temperature. On the third day, oat flakes were added directly onto an actively growing plasmodium on a nutrient agar plate and incubated for one additional day. At this point, a plug of agar (ca 2 cm^2^) bearing an active plasmodium from each of these plates was used as the primary inoculum. The cultures were incubated in the dark at room temperature for 5 days, after which the plasmodium in each plate was collected and weighed to obtain a value for biomass production.

### Effect of medium components on plasmodial growth and lipid production

The effects of three medium components—rice bran concentration (x_1_), yeast extract concentration (*x*_2_) and agar concentration (x_3_)—on DCW (Y_1_) and product yield (Y_2_) were investigated using the Box-Benken central composite (Expert design software version 9). The experimental design is outlined in Table 4.

**Table 4:** Outline of the experimental design and the results obtained

Run no.	Independent variable			Dependent variable	
	Rice bran (g/L)	Yeast extract (g/L)	Agar (g/L)	DCW (g/L)	Yield
	*x*_*1*_	*x*_*2*_	*x*_*3*_	*Y*_*1*_	*Y*_*2*_
1	50	2.5	15	6.23	0.12
2	50	2.5	10	6.51	0.13
3	25	7.5	15	6.02	0.24
4	37.5	5	12.5	7.30	0.19
5	25	2.5	10	6.21	0.25
6	25	7.5	10	5.04	0.20
7	25	2.5	15	6.26	0.25
8	50	7.5	10	6.14	0.12
9	37.5	5	12.5	7.13	0.19
10	37.5	5	12.5	7.12	0.19
11	50	7.5	15	5.88	0.12
12	37.5	5	12.5	6.91	0.18
13	37.5	5	8.29	5.66	0.15
14	37.5	5	16.70	6.77	0.18
15	58.5	5	12.5	6.81	0.12
16	37.5	5	12.5	7.24	0.19
17	37.5	0.79	12.5	7.52	0.20
18	16.4776	5	12.5	5.12	0.31
19	37.5	9.20	12.5	6.70	0.18
20	37.5	5	12.5	7.13	0.19

It should be noted that since rice bran is not soluble, each treatment was prepared by combining the appropriate amount of rice bran with 50 mL of the type of medium being tested. This was thoroughly mixed and then quickly poured into each large Petri dish (14 cm diam.) after being autoclaved. The Petri dish was swirled until the rice bran was evenly distributed throughout the plate.

### Effect of incubation time on plasmodial growth and lipid production

The optimized medium, determined from the experiments described above, was used for the rest of the study. Plasmodial cultures of *P. polycephalum* were prepared in 16 plates (14 cm diam.), each containing 50 ml of the optimized medium. The same medium and size of the plasmodial inoculum (ca 2 cm^2^) were used, and the cultures were incubated under the same conditions as noted above. Therefore, all the cultures would be expected to display the same growth profile. Two plates were selected each day for biomass collection and lipid analysis.

### Determination of dry cell weight

The fresh biomass of *P. polycephalum* was lyophilized (Labconco, freeze-zone six) to a constant weight and weighed on an analytical balance (AB104-S, Switzerland).

### Determination of total lipids

The amount of total lipids was determined with a modified Bligh-Dyer method. In brief, a ternary mixture of dichloromethane, methanol and water (1:2:0.8) was added to the freeze-dried sample powder. The addition of water to the sample was necessary to form the ternary mixture, as the sample had no water present. The resulting mixture of sample and solvent was vortexed and centrifuged [16]. The lower phase of the mixture containing the lipids was transferred to a pre-weighed glass vial and then dried under a N_2_ gas flow. The total amount of lipids in each sample was determined by using an analytical balance (AB104-S, Switzerland) [5].

### Determination of lipid content

Lipid content was determined as

$$ \mathrm{Lipid}\ \mathrm{content}\ \left(\%\right)=\frac{\mathrm{Lipid}\ \mathrm{amount}\ \left(\mathrm{g}\right)*100\%}{\mathrm{DCW}\ \left(1\mathrm{g}\right)} $$

where DCW is the dry cell weight.
